# Adolescent Cognitive Aptitudes and Later-in-Life Alzheimer Disease and Related Disorders

**DOI:** 10.1001/jamanetworkopen.2018.1726

**Published:** 2018-09-07

**Authors:** Alison R. Huang, Kiersten L. Strombotne, Elizabeth Mokyr Horner, Susan J. Lapham

**Affiliations:** 1 American Institutes for Research, Washington, DC

## Abstract

**Importance:**

Low early-life cognitive ability is a potential early marker of dementia risk in later life. Previous studies use only global measures of general intelligence and/or study this relationship in gender-specific samples. The contribution of early-life performance on specific cognitive abilities, such as language, reasoning, and visualization aptitudes, to indicating future dementia risk is unknown.

**Objectives:**

To investigate the association between adolescent cognitive ability and Medicare-recorded Alzheimer disease and related disorders (ADRD) using both general and specific measures of cognitive ability and to explore these associations separately in men and women.

**Design, Setting, and Participants:**

Population-based cohort study from the Project Talent–Medicare linked data set, a linkage of adolescent sociobehavioral data collected from high school students in 1960 to participants’ 2012 to 2013 Medicare Claims and expenditures data. The association between adolescent cognitive ability and risk of ADRD in later life was assessed in a diverse sample of 43 014 men and 42 749 women aged 66 to 73 years using a series of logistic regressions stratified by sex, accounting for demographic characteristics, adolescent socioeconomic status, and regional effects. Data analysis was conducted from November 2017 to March 2018.

**Main Outcomes and Measures:**

Presence of Medicare-reported ADRD.

**Results:**

Overall, 1239 men (2.9%) and 1416 women (3.3%) developed ADRD. Lower mechanical reasoning was associated with increased odds of ADRD in men (odds ratio, 1.17; 95% CI, 1.05-1.29), and lower memory for words in adolescence was associated with increased odds of ADRD in women (odds ratio, 1.16; 95% CI, 1.05-1.28). Lower performance on several other language, reasoning, visualization, and mathematic aptitudes in adolescence showed prominent, but weaker, associations with odds of ADRD.

**Conclusions and Relevance:**

This work contributes to the understanding of early-life origins of ADRD risk. The results suggest specific measures of cognitive ability may contribute to very early identification of at-risk subgroups who may benefit from prevention or intervention efforts.

## Key Points

**Question:**

What are the associations between specific adolescent cognitive abilities and Alzheimer disease and related disorders in later life?

**Findings:**

In this cohort study of 43 014 men and 42 749 women, lower adolescent memory for words, in women, and lower mechanical reasoning, in men, were associated with higher odds of Alzheimer disease and related disorders in later life.

**Meaning:**

Low performance on certain specific measures of cognitive ability may indicate future risk of Alzheimer disease and related disorders as early as adolescence.

## Teaser

This population-based cohort study investigates the association between adolescent cognitive ability and later-in-life Medicare-recorded Alzheimer disease and related disorders and explores the association seperately in men and women.

## Introduction

Dementia affects more than 50 million people globally and, as a major cause of disability and dependence, it is an overwhelming condition for patients and caregivers.^1^ Risk factors for dementia extend across the life course and are genetic, environmental, and social in nature.^2,3,4,5^ Based on the cognitive reserve hypothesis, high levels of cognitive functioning and reserve accumulated throughout the life course may protect against brain pathology and clinical manifestations of dementia.^6^ Several studies have observed a relationship between higher levels of early-life cognitive ability and lower risk of dementia.^7,8,9,10,11,12^ Notably, the Scottish Mental Health Survey^10^ demonstrated an association between lower mental ability at age 11 years and increased risk of dementia, and this relationship was stronger in women compared with men. Other studies have investigated this relationship in samples of only 1 sex.

In the Nun Study, higher linguistic ability measured from autobiographical essays written by young women was associated with reduced risk of cognitive impairment and Alzheimer disease (AD).^7^ Additionally, 2 large studies of young men^8,9^ showed associations of low cognitive ability with higher risk of dementia in later life. A strength of these studies is that with a relatively homogeneous sample of the same sex and similar life experience, investigators could account for much of the unobserved variance in this association. Additionally, there is evidence of differences between men and women in the influence of dementia risk factors and progression of disease,^13^ which motivates analyses stratified by sex. On the other hand, a more diverse sample allows for broader population relevance and the opportunity to examine the same risk factors by sex.

One commonality across studies of children and young adults is that investigators have used a global or combined score derived from individual tests to measure cognitive ability.^8,9^ The Scottish Mental Health Survey^10^ measured childhood intelligence with the Moray House Test, a general test of cognitive ability, while a study investigating dementia risk and cognitive reserve used a mean of school grades.^11^ The Nun Study tested the association of individual cognitive measures with cognition and AD in later life.^7^ However, this analysis was limited to 2 linguistic measures in a small sample of 93 women. To our knowledge, no studies have investigated this relationship using specific measures of early-life cognitive ability, likely because availability of early-life data is scarce in aging studies.

While measures of general cognition are undeniably important for understanding the broad-causal pathway between risk factors and disease, specific cognitive measures may be valuable for revealing particular areas of the brain affected by dementia. In adults, recall of organizable words, recognition of faces, and letter fluency are associated with AD risk even after accounting for performance on the Mini-Mental State Examination.^14^ Elucidating the specific early-life cognitive abilities that reliably correlate with dementia risk in later life can inform targeted focus areas for prevention and intervention efforts.

We used the Project Talent–Medicare (PT-MED) linked data to investigate the relationship between adolescent cognitive ability and Medicare-recorded AD and related disorders (ADRD) using both general and specific measures of cognition, and explored these relationships separately in men and women. We used specific measures of adolescent language ability, complex intellectual aptitude, visualization, mathematics, and clerical and perceptual aptitude. To our knowledge, this is the first study to investigate the relationship between a comprehensive set of adolescent cognitive aptitudes and Medicare-recorded ADRD in a large sample of both men and women.

## Methods

### Study Population

Data for this study came from PT-MED, a linkage of sociobehavioral data collected from high school students in 1960 to participants’ Medicare claims and expenditures data.^15^ Project Talent (PT), first conducted in 1960 (PT60), was a nationally representative study of 377 016 American high school students. The study was a stratified, random sample of 1225 secondary schools from all states except Alaska. Nearly all students in sampled schools completed the 1980 item assessment. In 2016, researchers linked 38% of the PT60 sample (n = 143 698) to their Medicare record and their 2012 to 2013 Medicare claims data. The current analyses used the 2013 Medicare claims data.^15^ Medicare, a US government program, provides health care to individuals aged 65 years and older and certain younger individuals.^16^ This study was approved by the institutional review board at the American Institutes for Research. The board approved a waiver of consent for this study. We followed the Strengthening the Reporting of Observational Studies in Epidemiology (STROBE) reporting guideline.

Of the 130 643 participants enrolled in Medicare in 2013, we excluded 44 880 participants from this analysis: 38 307 participants were enrolled in Medicare managed care plans (Part C), which provide benefits in addition to hospital and medical insurance (Parts A and B), and had incomplete claims data^17^; 6509 remaining participants did not provide credible responses to PT60; and 64 remaining participants were outside the expected age range (ages 13-20 years) for high school students. This brings the sample size for this analysis to 85 763 participants (43 014 men and 42 749 women).

Participants were matched to their Medicare record by their social security number, when available, or a combination of last name, date of birth, and sex.^15^ Compared with PT60, the analytic sample contained a larger proportion of follow-up survey respondents and smaller proportion of African American participants (eTable 1 in the Supplement). There were minimal differences in participant age, grade, sex, socioeconomic status (SES), region, and adolescent IQ. A larger proportion of follow-up survey respondents appeared in the analytic sample because social security numbers obtained through these collections facilitated record linkages. Compared with the 2013 Medicare population aged 65 to 74 years, the analytic sample had a larger proportion of white beneficiaries and smaller proportion of African American beneficiaries.^15^

### Measures of Adolescent Cognitive Ability

A comprehensive battery of adolescent cognitive aptitude tests were included in PT60 (Table 1).^18^ These included 2 measures of general cognitive ability, IQ and general academic aptitude, and 17 specific measures of cognitive aptitude. The specific measures included 5 language aptitudes and ability tests (memory for sentences, memory for words, disguised words, word functions in sentences, and reading comprehension), 3 complex intellectual aptitudes (creativity, mechanical reasoning, and abstract reasoning), 2 visualization tests (visualization in 2 dimensions and in 3 dimensions), 3 mathematics tests (arithmetic reasoning, introductory mathematics, and advanced mathematics), and 4 clerical and perceptual aptitudes (arithmetic computation, table reading, clerical checking, and object inspection).^18^ We calculated standardized z scores by sex for all measures. Cognitive scales were reverse coded in regression analyses such that an increase in score reflects an increase in disadvantage.

**Table 1.** Description of Project Talent Cognitive Aptitudes and Abilities^a^

Measure (No. of Items)	Description
General cognitive ability	
IQ composite (79)	Measures IQ through measures of reading comprehension, reasoning, and mathematics
General academic aptitude (329)	Measures general academic aptitude through measures of mathematics, English, vocabulary, and reasoning
Language aptitude and abilities	
Memory for sentences (16)	Measures a particular kind of memory—the ability to memorize simple descriptive statements and to recall a missing word when the rest of the sentence is provided sometime later
Memory for words (24 items)	Measures a type of rote memory—the ability to memorize foreign words corresponding to common English words
Disguised words (30)	Measures the ability to form connections between letters and sounds
Word function in sentences (24)	Measures the student’s sensitivity to grammatical structure; the fact that the terminology of grammar is not used at all in the test helps reduce the effects of formal training to a minimum; to score well, one must understand sentence structure and be able to recognize the function of each word or phrase in the sentence
Reading comprehension (48)	Measures the ability to comprehend written materials; the test includes passages on a wide range of topics; the student reads each passage and then answers a number of questions about it, referring back to the passage at will
Complex intellectual aptitude	
Creativity (20)	Measures the ability to find ingenious solutions to a variety of practical problems; items on this test require the student to generate tentative solutions and match them to multiple-choice alternatives indicated by a single letter of the solution word
Mechanical reasoning (20)	Measures the ability to deduce the effects of the operation of everyday physical forces (eg, gravity) and basic kinds of mechanisms (eg, gears, pulleys, wheels, springs, and levers)
Abstract reasoning (15)	A nonverbal test designed to measure the ability to determine a logical relationship or progression among the elements of a complex pattern and to apply this relationship to identify an element that belongs in a specified position in a pattern
Visualization	
Visualization in 2 dimensions (24)	Measures the ability to visualize how diagrams would look after being turned around on a flat surface, in contrast to the way they would look after being turned over
Visualization in 3 dimensions (16)	Measures the ability to visualize how a 2-dimensional figure would look after it had been folded to make a 3-dimensional figure
Mathematics	
Arithmetic reasoning (16)	Measures the ability to reason in the manner required to solve arithmetic problems; computation, except at the very simplest level, is excluded from the test
Introductory high school math (24)	Measures achievement in all kinds of mathematics generally taught up to and including ninth grade, with the exception of the areas covered in the Arithmetic Computation Test Arithmetic Reasoning Test; the primary emphasis of this test is on elementary algebra, and other topics include fractions, decimals, percentages, square roots, intuitive geometry, and elementary measurement formulas
Advanced high school math (14)	Measures understanding and application of basic concepts and methods rather than rote memory; the topics covered are plane geometry, solid geometry, algebra, trigonometry, elements of analytic geometry, and introductory calculus
Clerical and perceptual aptitudes	
Arithmetic computation (72)	Measures speed and accuracy of computation; the test is limited to the 4 basic operations (addition, subtraction, multiplication, and division) and to whole numbers
Table reading (72)	Measures speed and accuracy in a noncomputational clerical task, involving obtaining information from tables
Clerical checking (74)	Measures speed and accuracy of perception in a simple clerical task; the test involves comparing pairs of names to determine whether they are identical
Object inspection (40)	Measures speed and accuracy in perception of form; measures the ability to spot differences in small objects quickly and accurately when comparing them visually

^a^ Detailed descriptions of each measure can be found in *The Project Talent Data Bank Handbook*.^18^

### Measure of ADRD Outcome

The Centers for Medicare and Medicaid Services uses an algorithm based on Medicare fee-for-service claims diagnosis and procedures codes to identify individuals with ADRD and senile dementia. The Centers for Medicare and Medicaid Services defines an ADRD occurrence if an ADRD *International Classification of Disease, Ninth Revision* diagnosis code (codes 331.0, 331.11, 331.19, 331.2, 331.7, 290.0, 290.10, 290.11, 290.12, 290.13, 290.20, 290.21, 290.3, 290.40, 290.41, 290.42, 290.43, 294.0, 294.10, 294.11, 294.20, 294.21, 294.8, and 797) was reported on at least 1 inpatient, skilled nursing facility, home health agency, hospital outpatient, physician, or supplier claim during a 3-year reference period.^19^ Compared with clinical assessments, identification of AD occurrence through Medicare claims has a sensitivity and specify of 0.64 and 0.95, respectively. Sensitivity is 0.85 and specificity is 0.89 for dementia occurrence.^20^ We recoded the 2013 flag for the first occurrence of ADRD to create a binary outcome measure for condition diagnosis.

### Covariables

Birth year, race, and region of residence in 2013 came from the claims data. Adolescent SES and region of school in 1960 came from the PT60 data. Adolescent SES was a composite measure composed of 9 self-reported measures of household income, home environment, and maternal and paternal education.^18^ We calculated the standardized *z* score by sex for adolescent SES. Participants’ SES was reverse coded in regression analyses such that an increase in score reflected an increase in disadvantage.

### Statistical Analysis

We conducted bivariate analyses by ADRD outcome using χ^2^ tests for categorical variables and *t* tests for continuous variables. We then examined the association between adolescent cognitive ability with ADRD in later life using a series of logistic regression models. We stratified by sex, as previous research shows sex-related differences in dementia prevalence, progression, and risk factors.^13^ Stratification by sex also allowed results to be compared with studies using sex-specific samples. We adjusted these analyses for birth year, race, adolescent SES (interpreted per standard deviation [SD] disadvantage), region of school in 1960, and region of residence in 2013. Participants with missing cognitive aptitude scores were excluded from associated statistical models.

We expressed cognitive aptitude measures as *z* scores such that a 1-unit increase in odds ratio corresponds to a SD disadvantage in cognitive ability score. We used Bonferroni correction to reduce the likelihood of observing type I errors when conducting multiple regression analyses. We used a Bonferroni-corrected α (α = .001), calculated by dividing our original *P* value (.05) by the number of regression analyses conducted (n = 38), and present Bonferroni-corrected 95% simultaneous confidence intervals. All standard errors are clustered by school attended in 1960. All analyses were conducted using STATA statistical software, version 13.1 (StataCorp LP).

## Results

In a sample of 43 014 men and 42 749 women, incidence of Medicare-reported ADRD was 2.9% in men (n = 1239) and 3.3% in women (n = 1416) (Table 2). This was slightly lower than the rate of ADRD (4% in both men and women) in the national Medicare population, aged 65 to 74 years,^15,21^ likely because this sample was slightly younger (aged 66 to 73 years in 2013) and had a smaller proportion of African American individuals, who are at higher risk of ADRD compared with white individuals.^22,23,24^ In bivariate analysis, participants significantly differed by ADRD outcome in birth year, race, region of school in 1960, and SES. Additionally, we observed significant differences by ADRD outcome for all cognitive aptitudes, except table reading and clerical checking. Advanced high school mathematics and object inspection aptitudes were significantly different by ADRD outcomes in women only.

**Table 2.** Demographic Characteristics and Cognitive Aptitude Scores by ADRD Outcome

Demographic Characteristic	Men		Women	
	No ADRD (n = 41 775)	ADRD (n = 1239)	No ADRD (n = 41 333)	ADRD (n = 1416)
Birth year, No. (%)^a^^,^^b^				
1940	236 (0.6)	14 (1.1)	108 (0.3)	6 (0.4)
1941	1534 (3.7)	85 (6.9)	908 (2.2)	50 (3.5)
1942	8852 (21.2)	363 (29.3)	8713 (21.1)	380 (26.8)
1943	10 172 (24.3)	321 (25.9)	10 019 (24.2)	412 (29.1)
1944	10 895 (26.1)	254 (20.5)	10 801 (26.1)	305 (21.5)
1945	9135 (21.9)	188 (15.2)	9694 (23.5)	245 (17.3)
1946	890 (2.1)	14 (1.1)	1017 (2.5)	17 (1.2)
1947	61 (0.1)	0	73 (0.2)	1 (0.1)
Race, No. (%)^a^^,^^b^				
White	39 166 (93.8)	1150 (92.8)	38 263 (92.6)	1271 (89.8)
Black	990 (2.4)	52 (4.2)	1660 (4.0)	103 (7.3)
Other	1196 (2.9)	30 (2.4)	1213 (2.9)	41 (2.9)
Missing	423 (1.0)	7 (0.6)	197 (0.5)	1 (0.1)
School region in 1960, No. (%)^a^^,^^b^				
Northeast	12 867 (30.8)	367 (29.6)	12 242 (29.6)	388 (27.4)
Midwest	14 220 (34.0)	410 (33.1)	14 076 (34.1)	476 (33.6)
South	10 559 (25.3)	349 (28.2)	10 882 (26.3)	446 (31.5)
West	4117 (9.9)	112 (9.0)	4120 (10.0)	105 (7.4)
Missing	12 (0)	1 (0.1)	13 (0)	1 (0.1)
Region of residence in 2013, No. (%)^b^				
Northeast	8643 (20.7)	279 (22.5)	8696 (21.0)	287 (20.3)
Midwest	9257 (22.2)	299 (24.1)	9479 (22.9)	343 (24.2)
South	15 393 (36.8)	469 (37.9)	15 620 (37.8)	593 (41.9)
West	7168 (17.2)	160 (12.9)	6440 (15.6)	161 (11.4)
Other	1314 (3.1)	32 (2.6)	1097 (2.7)	32 (2.3)
Missing	0	0	1 (0)	0
Socioeconomic status (0-135), mean (SD)^a^^,^^b^	99.9 (9.8)	99.2 (10.0)	99.7 (9.5)	98.6 (9.6)
Missing, No. (%)	1882 (4.5)	51 (4.1)	1492 (3.6)	60 (4.2)
Cognitive aptitudes and abilities				
General intelligence				
IQ (0-293), mean (SD)^a^^,^^b^	182.6 (49.4)	175.4 (51.6)	180.1 (48.2)	172.4 (51.0)
Missing, No. (%)	851 (2.0)	18 (1.5)	745 (1.8)	30 (2.1)
General academic ability (0-829), mean (SD)^a^^,^^b^	531.2 (115.2)	514.9 (118.0)	532.4 (106.1)	513.6 (112.1)
Missing, No. (%)	1857 (4.4)	54 (4.4)	1660 (4.0)	61 (4.3)
Language aptitude and abilities				
Memory for sentences (0-16), mean (SD)^a^^,^^b^	9.0 (2.9)	8.8 (3.1)	9.8 (3.0)	9.5 (3.1)
Missing, No. (%)	732 (1.8)	16 (1.3)	633 (1.5)	25 (1.8)
Memory for words (0-24), mean (SD)^a^^,^^b^	11.5 (5.2)	10.7 (4.9)	13.3 (5.5)	12.5 (5.6)
Missing, No. (%)	731 (1.7)	17 (1.4)	633 (1.5)	25 (1.8)
Disguised words (0-30), mean (SD)^a^^,^^b^	15.5 (6.7)	14.7 (6.7)	16.8 (6.8)	16.0 (6.9)
Missing, No. (%)	770 (1.8)	18 (1.5)	664 (1.6)	26 (1.8)
Word function in sentences (0-24), mean (SD)^a^^,^^b^	10.4 (5.3)	9.8 (5.1)	12.0 (5.7)	11.2 (5.8)
Missing, No. (%)	789 (1.9)	19 (1.5)	669 (1.6)	27 (1.9)
Reading comprehension (0-48), mean (SD)^a^^,^^b^	32.3 (10.0)	31.1 (10.4)	32.9 (9.5)	31.7 (9.8)
Missing, No. (%)	759 (1.8)	18 (1.5)	654 (1.6)	27 (1.9)
Complex intellectual aptitude				
Creativity (0-20), mean (SD)^a^^,^^b^	9.9 (4.0)	9.6 (4.1)	9.0 (3.7)	8.6 (3.7)
Missing, No. (%)	792 (1.9)	18 (1.5)	693 (1.7)	26 (1.8)
Mechanical reasoning (0-20), mean (SD)^a^^,^^b^	13.2 (3.8)	12.6 (4.1)	9.0 (3.5)	8.6 (3.5)
Missing, No. (%)	791 (1.9)	18 (1.5)	681 (1.6)	27 (1.9)
Abstract reasoning (0-15), mean (SD)^a^^,^^b^	9.6 (2.8)	9.2 (2.9)	9.3 (2.8)	8.9 (3.0)
Missing, No. (%)	834 (2.0)	19 (1.5)	738 (1.8)	32 (2.3)
Visualization				
Visualization in 2 dimensions (0-24), mean (SD)^a^^,^^b^	14.5 (5.5)	13.7 (5.7)	12.3 (5.4)	11.7 (5.5)
Missing, No. (%)	802 (1.9)	19 (1.5)	689 (1.7)	28 (2.0)
Visualization in 3 dimensions (0-16), mean (SD)^a^^,^^b^	9.5 (3.3)	9.1 (3.5)	8.4 (3.0)	8.1 (3.0)
Missing, No. (%)	828 (2.0)	20 (1.6)	733 (1.8)	32 (2.3)
Mathematics				
Arithmetic reasoning (0-16), mean (SD)^a^^,^^b^	9.4 (3.5)	9.1 (3.6)	8.7 (3.4)	8.2 (3.5)
Missing, No. (%)	784 (1.9)	18 (1.5)	707 (1.7)	30 (2.1)
Introductory high school math (0-24), mean (SD)^a^^,^^b^	12.1 (5.0)	11.4 (5.1)	11.0 (4.6)	10.3 (4.6)
Missing, No. (%)	777 (1.9)	18 (1.5)	705 (1.7)	29 (2.0)
Advanced high school math (0-14), mean (SD)^a^	3.8 (2.7)	3.7 (2.7)	3.0 (2.2)	2.9 (2.0)
Missing, No. (%)	1084 (2.6)	21 (1.7)	1036 (2.5)	43 (3.0)
Clerical and perceptual aptitudes				
Arithmetic computation (0-72), mean (SD)^a^^,^^b^	38.9 (9.6)	37.8 (9.7)	40.6 (9.4)	39.2 (10.1)
Missing, No. (%)	1033 (2.5)	23 (1.9)	832 (2.0)	35 (2.5)
Table reading (0-72), mean (SD)	12.9 (8.3)	12.7 (7.8)	13.2 (6.9)	12.9 (7.3)
Missing, No. (%)	1013 (2.4)	22 (1.8)	802 (1.9)	31 (2.2)
Clerical checking (0-74), mean (SD)	36.3 (14.2)	36.7 (14.7)	39.4 (13.5)	39.5 (14.3)
Missing, No. (%)	1067 (2.6)	26 (2.1)	821 (2.0)	34 (2.4)
Object inspection (0-40), mean (SD)^b^	23.0 (7.2)	22.8 (7.4)	24.0 (6.6)	23.5 (6.8)
Missing, No. (%)	986 (2.4)	21 (1.7)	783 (1.9)	33 (2.3)

Abbreviation: ADRD, Alzheimer disease and related disorders.

^a^ Significant difference (*P* < .05) in ADRD outcome in men.

^b^ Significant difference (*P* < .05) in ADRD outcome in women.

We first present the association of 2 measures of general intelligence, IQ and general academic aptitude, with ADRD in later life (Table 3). Results from logistic regressions are expressed as odds ratios and Bonferroni-corrected 95% simultaneous confidence intervals. We express cognitive aptitude measures as z scores such that change in odds ratio (OR) should be interpreted per SD disadvantage in cognitive ability. Using a Bonferroni-corrected α, low IQ (men: OR, 1.17; 95% CI, 1.04-1.32; women: OR, 1.17; 95% CI, 1.04-1.31) and low general academic aptitude (men: OR, 1.18; 95% CI, 1.05-1.33; women: OR, 1.19; 95% CI, 1.06-1.33) were significantly associated with increased odds of ADRD in later life in both men and women.

**Table 3.** Association of General and Specific Adolescent Cognitive Aptitudes and Odds of Medicare-Recorded Alzheimer Disease and Related Disorders

Variables	Men			Women		
	No.	OR (95% CI)^a^	*P* Value^b^	No.	OR (95% CI)^a^	*P* Value^b^
General intelligence						
IQ	40 329	1.17 (1.04-1.32)	<.001	40 567	1.17 (1.04-1.31)	<.001
General Academic Aptitude	39 463	1.18 (1.05-1.33)	<.001	39 769	1.19 (1.06-1.33)	<.001
Language aptitude and abilities						
Memory for sentences	40 371	1.06 (0.96-1.18)	.06	40 615	1.07 (0.97-1.19)	.02
Memory for words	40 371	1.16 (1.05-1.27)	<.001	40 615	1.16 (1.05-1.28)	<.001
Disguised words	40 355	1.11 (1.00-1.23)	.001	40 593	1.12 (1.02-1.24)	<.001
Word functions in sentences	40 339	1.13 (1.03-1.25)	<.001	40 583	1.14 (1.03-1.27)	<.001
Reading comprehension	40 364	1.15 (1.02-1.29)	<.001	40 596	1.14 (1.02-1.27)	<.001
Complex intellectual aptitudes						
Creativity	40 348	1.09 (0.97-1.21)	.01	40 575	1.09 (0.99-1.20)	.005
Mechanical reasoning	40 351	1.17 (1.05-1.29)	<.001	40 586	1.09 (0.99-1.20)	.002
Abstract reasoning	40 337	1.12 (1.01-1.24)	<.001	40 567	1.13 (1.01-1.25)	<.001
Visualization						
Visualization in 2 dimensions	40 331	1.13 (1.03-1.24)	<.001	40 572	1.09 (0.99-1.20)	.005
Visualization in 3 dimensions	40 343	1.13 (1.02-1.25)	<.001	40 571	1.08 (0.99-1.19)	.005
Mathematics						
Arithmetic reasoning	40 361	1.12 (1.00-1.26)	.001	40 594	1.12 (1.02-1.24)	<.001
Introductory math	40 365	1.15 (1.03-1.28)	<.001	40 598	1.11 (1.01-1.22)	<.001
Advanced math	40 104	1.12 (1.01-1.25)	<.001	40 300	1.04 (0.95-1.14)	.13
Clerical and perceptual aptitudes						
Arithmetic computation	40 146	1.15 (1.04-1.27)	<.001	40 464	1.14 (1.03-1.26)	<.001
Table reading	40 189	1.06 (0.95-1.19)	.08	40 504	1.07 (0.95-1.21)	.05
Clerical checking	40 128	0.99 (0.89-1.10)	.73	40 481	1.02 (0.91-1.14)	.50
Object inspection	40 219	1.04 (0.94-1.15)	.19	40 522	1.09 (0.99-1.20)	.003

Abbreviation: OR, odds ratio.

^a^ The ORs were estimated from logistic regression models controlled for birth year, race, adolescent socioeconomic status, region of school in 1960, and region of residence in 2013.

^b^ Bonferroni-corrected α set at .001; Bonferroni-corrected 95% simultaneous confidence intervals.

We examined the associations between each of 17 specific measures of adolescent cognitive aptitude and ADRD in later life using a series of logistic regression models stratified by sex and adjusted for birth year, race, adolescent SES (interpreted per SD disadvantage), region of school in 1960, and region of residence in 2013 (34 total regressions) (Table 3). In women, low memory for words in adolescence showed the strongest association with ADRD in later life such that 1 SD disadvantage was associated with 1.16-fold increased odds (OR, 1.16; 95% CI, 1.05-1.28). In men, low memory for words was also an important indicator (OR, 1.16; 95% CI, 1.05-1.27); however, mechanical reasoning showed a slightly more robust association; 1 SD disadvantage in mechanical reasoning was associated with 1.17-fold higher odds of ADRD (OR, 1.17; 95% CI, 1.05-1.29). Additionally, lower performance in word function in sentences (men: OR, 1.13; 95% CI, 1.03-1.25); women: OR, 1.14; 95% CI, 1.03-1.27), reading comprehension (men: OR, 1.15; 95% CI, 1.02-1.29; women: OR, 1.14; 95% CI, 1.02-1.27), abstract reasoning (men: OR, 1.12; 95% CI, 1.01-1.24; women: OR, 1.13; 95% CI, 1.01-1.25), introductory math (men: OR, 1.15; 95% CI, 1.03-1.28; women: OR, 1.11; 95% CI, 1.01-1.22), and arithmetic computation (men: OR, 1.15; 95% CI, 1.04-1.27; women: OR, 1.14; 95% CI, 1.03-1.26) was associated with increased odds of ADRD in both men and women. Visualization in 2 dimensions (OR, 1.13; 95% CI, 1.03-1.24), visualization in 3 dimensions (OR, 1.13; 95% CI, 1.02-1.25), and advanced math (OR, 1.12; 95% CI, 1.01-1.25) in men, and disguised words (OR, 1.12; 95% CI, 1.02-1.24) and arithmetic reasoning (OR, 1.12; 95% CI, 1.02-1.24) in women, were also prominent factors in this association.

We also investigated a nonlinear association between adolescent cognitive abilities and ADRD by modeling cognitive abilities in terciles, with highest tercile of ability as the reference category (eTable 2 in the Supplement). We observed similar results in models presented in Table 3. Where differences were significant, health increased monotonically, with individuals in the lowest tercile exhibiting significant disadvantage compared with those in the highest tercile. Further discussion and differences are highlighted in eTable 2 in the Supplement.

While the associations between cognitive aptitudes and ADRD did appear to differ between men and women, sex by cognitive ability interaction analyses on the pooled sample showed these differences were not statistically significant. Thus, sex differences should not be overinterpreted. We present stratified analyses because genetic, biological, and psychosocial ADRD risk factors affect men and women differently.^13^ Stratified results also allow the current results to be compared with studies using only sex-specific samples.

## Discussion

### Main Findings and Comparison With Other Studies

We found that multiple specific, adolescent cognitive aptitudes were associated with ADRD 53 years after initial testing in high school. Lower mechanical reasoning and memory for words in adolescence showed the strongest associations with increased odds of ADRD in men and women, respectively. These findings were independent of birth cohort, race, adolescent SES, and regional effects. While this sample was relatively young for ADRD (aged 66-73 years in 2013), this study provides a first glance into the relationship between adolescent cognitive aptitudes and ADRD in this cohort.

Our findings regarding IQ and general academic aptitude are consistent with previous studies.^7,8,9,10,11^ In this study, the magnitude of the association was similar in men and women in contrast to findings from the Scottish Mental Health Survey. Russ et al^10^ found a significant increase in dementia risk per SD of IQ disadvantage at age 11 years in women (IQ disadvantage: hazard ratio per SD, 1.14; 95% CI, 1.09-1.19); however, a significant association was not observed in men (IQ disadvantage: hazard ratio per SD, 1.02; 95% CI, 0.97-1.08).

We extend previous research by investigating the specific adolescent cognitive abilities associated with future ADRD. One other study (the Nun Study) has explored the contribution of specific early-life cognitive abilities by analyzing participant written autobiographies,^7^ likely because the availability of this kind of data in conjunction with later-life data are rare. We found lower memory for words aptitude in adolescence was associated with increased risk of ADRD in both men and women, consistent with research on verbal memory in adulthood. Verbal memory is independently associated with dementia 3 to 8 years after baseline measurement in healthy adults.^14,25,26,27^ Poor performance on verbal memory tests was also associated with conversion to AD in adults with mild cognitive impairment.^28,29^ Our findings demonstrate that this relationship extends as far back as adolescence.

Our results support the Nun Study’s findings that linguistic aptitude in young adulthood is strongly associated with cognitive decline and AD in women.^7^ Lower performance in all PT60 language aptitudes, except memory for sentences, are prominent indicators associated with higher ADRD risk in women. However, PT-MED differs from the Nun Study in sample composition and cognitive measures. Compared with women in the Nun Study, PT60 participants come from more diverse backgrounds, were born more than 20 years later, and are subject to more heterogeneous life experiences. Furthermore, PT60 uses aptitude measures based on a series of objective questions, while the Nun Study derived linguistic ability indicators from participant written autobiographies.

In adults, arithmetic computation, mechanical and abstract reasoning, and visuospatial functioning are discriminative indicators in dementia progression. Arithmetic abilities, such as arithmetic operations and calculations, are associated with financial capacity, a skill sensitive to dementia and AD pathology.^30^ Additionally, the Bicycle Drawing Test, a measure of mechanical reasoning and visuographic functioning, is suggested as a good indicator of cognitive decline.^31^ There are, however, mixed findings regarding visuospatial functioning.^14,27,31^ In healthy older adults, lower abstract reasoning scores are associated with probable AD 10 years later.^32^ We show that measures of arithmetic computation, mechanical and abstract reasoning, and visualization, which are evident indicators in adults, are also robust indicators associated with ADRD decades earlier.

### Limitations

The ADRD cases were identified through Medicare claims, not through a clinical assessment. Medicare claims have a sensitivity and specificity of 0.64 and 0.95 for AD and 0.85 and 0.89 for dementia, respectively, compared with clinical assessment.^20^ Claims data also show reasonable consistency with cases clinically identified in an AD registry.^33^ However, patients with mild or early-stage ADRD may be missed because physicians are less likely to code individuals using ADRD codes.^33^ Additionally, patients who have not yet obtained medical attention will be missed altogether. This suggests that our results may be an underestimate of the association and may reflect individuals more severely affected by ADRD.

Characteristics of the sample may also introduce bias. The lower proportion of African American beneficiaries, who are at higher risk for ADRD, in the analytic sample suggests negative bias.^22,23,24^ Little is known about ADRD risk in Medicare Part C beneficiaries and the potential biases introduced by exclusion of this group. However, in this sample, Medicare Part C beneficiaries had similar distributions of participant characteristics but marginally lower adolescent cognitive scores compared with fee-for-service beneficiaries. This limitation is endemic to any study using Medicare claims as claims data are incomplete for Part C enrollees.^17^ Potential midlife mediators are not included in this analysis. This study focuses on the contribution of adolescent factors; however, data currently being collected for the 2018 PT Aging Study may provide opportunities to expand this work. Additionally, in the future, the American Institutes for Research plans to link the full PT60 sample to Medicare records. As our cohort ages and additional years of data become available, we can use methods such as Cox proportional hazards to further specify the association between adolescent cognitive abilities and ADRD.

### Possible Mechanisms and Implications

The mechanism underlying the relationship between adolescent cognitive ability and ADRD is still unclear; however, these results provide insight into the specific aspects of cognitive aptitude involved. One explanation is that poor performance on cognitive tests in adolescence reflects poor brain development earlier in life, a risk factor for dementia.^34^ Lower cognitive ability in adolescence may also identify individuals at risk for low accumulation of cognitive reserve throughout life. Based on the cognitive reserve hypothesis, individuals with low levels of accumulated reserve are less prepared to deal with neuropathology and are more at risk for cognitive impairment in later life.^6^ Alternatively, cognitive ability in early life may be an indicator of health behaviors in adulthood.^35^ Poor health behaviors, such as smoking and physical inactivity, are risk factors for ADRD.^36,37^ Regardless of mechanism, our findings emphasize that early-life risk stretches across the life course.

These findings have implications for dementia and AD identification and prevention. Children with known ADRD risk already exhibit certain differences in brain structure,^34,38,39^ and understanding ADRD-associated functional impairments along detailed lines of cognition may suggest specific brain networks for further investigation of early pathology. As more attention and research are devoted to very early detection^40^ and premorbid cognitive interventions,^41,42^ performances on specific cognitive aptitudes could eventually contribute to identification, as early as adolescence, of at-risk subgroups for which intervention efforts would be especially beneficial. Efforts to promote cognitive reserve–building experiences and positive health behaviors throughout the life course may prevent or delay clinical symptoms of ADRD in later life.^6,36,37^ Postponement or prevention of ADRD would have substantial societal impacts considering the large social and economic consequences these conditions have for patients, caregivers, and the health care system.

## Conclusions

In sum, lower performance on certain specific measures of cognitive ability in adolescence are associated with increased likelihood of ADRD 53 years later. Low mechanical reasoning and memory for words show the strongest associations with odds of ADRD in men and women, respectively. Several other language, reasoning, visualization, and mathematic aptitudes are also prominent factors in this association. Our results support the early-life origins of ADRD risk and bring forth the potential for specific measures of cognitive ability to assist in very early identification of subgroups at risk for ADRD.

## Notes

Supplement. **eTable 1.** Comparison of Distribution of Participant Characteristics in the Analytic Sample and PT60 **eTable 2.** Association of General and Specific Adolescent Cognitive Aptitudes (terciles) and Odds of Medicare-Recorded ADRD **eReference**
