# Supplementary material for: Adolescent Cognitive Aptitudes and Later-in-Life Alzheimer Disease and Related Disorders
Source: JAMA Netw Open. 2018 Sep 7;1(5):e181726. doi: 10.1001/jamanetworkopen.2018.1726 (PMC6324501; doi:10.1001/jamanetworkopen.2018.1726)
Supplement: Supplement. — eTable 1. Comparison of Distribution of Participant Characteristics in the Analytic Sample and PT60 eTable 2. Association of General and Specific Adolescent Cognitive Aptitudes (terciles) and Odds of Medicare-Recorded ADRD eReference [file jamanetwopen-1-e181726-s001.pdf]

Supplementary Online Content

Huang AR, Strombotne KL, Horner EM, Lapham SJ. Adolescent cognitive aptitudes and later-in-life Alzheimer disease and related disorders. *JAMA Netw Open*. 2018:1(5):e181726. doi:10.1001/jamanetworkopen.2018.1726

eTable 1. Comparison of Distribution of Participant Characteristics in the Analytic Sample and PT60

eTable 2. Association of General and Specific Adolescent Cognitive Aptitudes (terciles) and Odds of Medicare-Recorded ADRD

eReference

This supplementary material has been provided by the authors to give readers additional information about their work.

© 2018 Huang AR et al. *JAMA Network Open*.

eTable 1. Comparison of Distribution of Participant Characteristics in the Analytic Sample and PT60[a]

	%			SMD[≠]
	**Analytic Sample (**n=85 763)	**PT60 (**n=377 016)	**Difference**	
Age in 2013, y				0.08
<65	0	0	0	
65-68	25	22	3	
69-70	50.2	50.9	-0.7	
≥71	24.8	27.1	-2.3	
Grade in 1960				0.05
Grade 9	26.8	27.6	0	
Grade 10	26.4	26.4	-0.7	
Grade 11	23.3	24.5	0	
Grade 12	23.4	21.5	-1.2	
Sex				0
Male	50.2	49.9	0.2	
Female	49.8	50.1	0.1	
Race [a]				0.23
White	94.7	89.7	5.1	
Black/African American	3	7.2	-4.1	
Hispanic	0.4	1.6	-1.1	
Asian	0.7	0.6	0.1	
Native American	0.3	0.3	0	
Other	0.8	0.7	0.2	
Socioeconomic status, terciles				0.19
Lowest tercile	24.3	31.7	-7.4	
Middle tercile	34.3	32.6	1.7	
Highest tercile	37.3	30.6	6.6	
Missing	4.1	5	-1	
Geographic Region				0.13
Northeast	30.2	26.2	4	
Midwest	34	32.2	1.8	
South	25.9	29.2	-3.3	
West	9.9	12.3	-2.5	
Missing	0	0	0	
Population of area of school				0.08
Rural	15.4	13.1	2.3	
Suburban	55.9	57.6	-1.7	
Urban	9.1	10.7	-1.6	
Missing	19.7	18.7	1	
Response to 1- or 5-year follow-up survey				0.75
Responded	89.3	58.5	30.9	
Did not respond	10.7	41.5	-30.9	

© 2018 Huang AR et al. *JAMA Network Open*.

Adolescent IQ, terciles				
Lowest tercile	33.1	32.6	0.5	0.06
Middle tercile	32.9	32.7	0.1	
Highest tercile	32.1	31.8	0.3	
Missing	1.9	2.9	-0.9	

Abbreviations: PT60, Project Talent 1960 data collection; SMD, Standardized mean difference

[a] Because PT60 did not include self-reported race, a constructed race variable was created using data collected from recent Project Talent studies and administrative data linkages, school racial composition, and race reported by participants in the same family.

≠SMD > 0.20 was considered a potential source of bias.

We assessed the distribution of participant characteristics in the analytic sample and in PT60 (eTable 1). The differences between these samples were evaluated using the standardized mean difference (SMD). Variables with SMD greater than 0.20[1] were considered a potential source of bias. All SMDs in participant characteristics were smaller than 0.20 except for follow-up survey status (SMD = 0.70) and participant race (SMD=0.23).

We investigated the non-linear association between adolescent cognitive abilities and odds of ADRD in later life using terciles of cognitive aptitudes (eTable 2). The relationships appear monotonic, with individuals in the lowest tercile exhibiting significant disadvantage compared to those in the highest tercile. The magnitude of these associations was attenuated for individuals in the middle tercile, which in most cases was not significantly different from the highest tercile. As in the linear model, women's odds for developing ADRD were best predicted by memory for words. Among men, in this specification, odds of developing ADRD were best predicted by word function in sentences, followed by mechanical reasoning; for reference, linear models depict mechanical reasoning with the strongest association (Table 3 in main article). This suggests that, in men, both abilities are robust predictors, however odds are especially elevated for individuals in the lowest tercile of word function in sentences.

© 2018 Huang AR et al. *JAMA Network Open*.

eTable 2. Association of General and Specific Adolescent Cognitive Aptitudes (terciles) and Odds of Medicare-Recorded ADRD

	Men				Women			
	n	Highest Tercile	OR (95% CI)[a,b]		n	Highest Tercile	OR (95% CI)[a,b]	
			Middle Tercile	Lowest Tercile			Middle Tercile	Lowest Tercile
General Intelligence								
IQ	40,329	ref.	1.05 (0.82 - 1.35)	1.35*** (1.04 - 1.76)	40,567	ref.	1.11 (0.88 - 1.40)	1.35*** (1.05 - 1.74)
General Academic Aptitude	39,463	ref.	1.08 (0.83 - 1.40)	1.32*** (1.01 - 1.74)	39,769	ref.	1.08 (0.86 - 1.34)	1.36*** (1.06 - 1.76)
Language Aptitude and Abilities								
Memory for sentences	40,371	ref.	0.91 (0.71 - 1.18)	1.09 (0.87 - 1.38)	40,615	ref.	1.05 (0.84 - 1.33)	1.16 (0.91 - 1.47)
Memory for words	40,371	ref.	1.22 (0.93 - 1.60)	1.31*** (1.02 - 1.67)	40,615	ref.	1.10 (0.86 - 1.40)	1.41*** (1.12 - 1.77)
Disguised words	40,355	ref.	1.01 (0.78 - 1.31)	1.27*** (1.00 - 1.62)	40,593	ref.	1.21 (0.95 - 1.55)	1.28*** (1.00 - 1.63)
Word functions in sentences	40,339	ref.	1.25 (0.98 - 1.59)	1.35*** (1.04 - 1.76)	40,583	ref.	1.06 (0.82 - 1.37)	1.37*** (1.06 - 1.75)
Reading comprehension	40,364	ref.	1.20 (0.93 - 1.53)	1.33*** (1.01 - 1.76)	40,596	ref.	1.16 (0.91 - 1.47)	1.33*** (1.02 - 1.74)
Complex Intellectual Aptitudes								
Creativity	40,348	ref.	1.01 (0.78 - 1.31)	1.17 (0.89 - 1.54)	40,575	ref.	1.30*** (1.03 - 1.65)	1.31*** (1.00 - 1.71)
Mechanical reasoning	40,351	ref.	1.18 (0.91 - 1.52)	1.31*** (1.03 - 1.67)	40,586	ref.	1.04 (0.83 - 1.30)	1.13 (0.90 - 1.41)

© 2018 Huang AR et al. *JAMA Network Open*.

Abstract reasoning	40,337	ref.	1.12 (0.85 - 1.46)	1.23 (0.97 - 1.56)	40,567	ref.	1.03 (0.80 - 1.32)	1.28 (0.98 - 1.69)
Visualization								
Visualization in 2D	40,331	ref.	1.09 (0.83 - 1.42)	1.34*** (1.05 - 1.70)	40,572	ref.	1.16 (0.91 - 1.49)	1.24 (0.97 - 1.59)
Visualization in 3D	40,343	ref.	1.03 (0.79 - 1.35)	1.20 (0.94 - 1.54)	40,571	ref.	1.06 (0.82 - 1.36)	1.21 (0.96 - 1.54)
Mathematics								
Arithmetic reasoning	40,361	ref.	1.07 (0.83 - 1.38)	1.18 (0.92 - 1.53)	40,594	ref.	1.20 (0.93 - 1.54)	1.27*** (1.01 - 1.62)
Introductory math	40,365	ref.	1.13 (0.87 - 1.46)	1.28 (0.99 - 1.67)	40,598	ref.	1.23 (0.94 - 1.59)	1.30 (1.00 - 1.68)
Advanced math	40,104	ref.	1.15 (0.88 - 1.51)	1.34*** (1.03 - 1.75)	40,300	ref.	1.09 (0.84 - 1.41)	1.08 (0.83 - 1.41)
Clerical and perceptual aptitudes								
Arithmetic computation	40,146	ref.	1.11 (0.86 - 1.43)	1.29*** (1.00 - 1.66)	40,464	ref.	1.10 (0.84 - 1.44)	1.35*** (1.05 - 1.73)
Table reading	40,189	ref.	0.92 (0.72 - 1.18)	1.07 (0.84 - 1.36)	40,504	ref.	1.02 (0.79 – 1.32)	1.18 (0.94 - 1.49)
Clerical checking	40,128	ref.	0.95 (0.74 - 1.21)	0.97 (0.76 - 1.26)	40,481	ref.	1.04 (0.83 - 1.30)	1.10 (0.86 - 1.39)
Object inspection	40,219	ref.	1.06 (0.81 - 1.37)	1.10 (0.85 - 1.41)	40,522	ref.	0.97 (0.77 - 1.22)	1.17 (0.93 - 1.47)

Abbreviations: OR, odds ratio; CI, confidence interval.

[a]Odds ratios estimated from logistic regression models controlled for birth year, race, adolescent socioeconomic status, region of school in 1960, and region of residence in 2013.

[b]Bonferroni-corrected alpha set at 0.001; Bonferroni corrected 95% simultaneous confidence intervals.

***p-value<0.001.

© 2018 Huang AR et al. *JAMA Network Open*.

eReference

1. Cohen J. Statistical power analysis for the behavioral sciences. 2nd. Hillsdale, NJ: Erlbaum; 1988.

© 2018 Huang AR et al. *JAMA Network Open*.
